# *Actinomycetota*-Derived Metabolites in Pancreatic Cancer: Chemical Diversity, Metabolic Vulnerability, and Translational Barriers

**DOI:** 10.1007/s11912-026-01842-y

**Published:** 2026-10-14

**Authors:** Daynara da Silva Pereira Lima, Marcelly da Silveira Silva, Moisés da Rocha Soares, Lucas Evangelista Lima Terceiro, Natanael Teles Ramos de Lima, Genil Dantas de Oliveira, Thalisson Amorim de Souza, Valnês Silva Rodrigues-Junior, Samuel Paulo Cibulski

**Affiliations:** 1 Programa de Pós-Graduação em Ciências Farmacêuticas, Departamento de Farmácia, Universidade Estadual da Paraíba (UEPB), Campina Grande, 58429-500 PB Brazil; 2 NuBioCaatinga. Núcleo de Bioprospecção da Caatinga, Universidade Federal do Rio Grande do Norte (UFRN), Santa Cruz, 59200-000 RN Brazil; 3 Department of Cellular and Physiological Sciences, Faculty of Medicine, University of British Columbia, Vancouver, V6T 1Z3 BC Canada; 4 Programa de Pós-Graduação em Produtos Naturais e Sintéticos Bioativos, Universidade Federal da Paraíba (UFPB), João Pessoa, 58051-900 PB Brazil; 5 CiTCoM UMR 8038, Faculté de Santé, Université Paris Cité, Paris, France; 6 Instituto de Ciências Biológicas e da Saúde (ICBS), Universidade Federal de Alagoas (UFAL), Maceió, 57072-970 AL Brazil; 7 FACISA - Faculdade de Ciências da Saúde do Trairi, Universidade Federal do Rio Grande do Norte (UFRN), Santa Cruz, 59200-000 RN Brazil

**Keywords:** Pancreatic ductal adenocarcinoma, *Streptomyces*, Microbial natural products, Cytotoxicity, Drug discovery

## Abstract

**Purpose of Review:**

Pancreatic ductal adenocarcinoma remains among the deadliest malignancies worldwide, and effective therapies are still lacking. Actinomycetota are among the richest microbial sources of bioactive secondary metabolites, and three agents in clinical use against pancreatic malignancy derive from them — streptozotocin, everolimus and doxorubicin — though all three are indicated for neuroendocrine rather than ductal tumors. This review provides a critical narrative synthesis of Actinomycetota-derived metabolites evaluated in pancreatic cancer models, with every potency value re-derived from the primary literature and interpreted alongside assay conditions and available mechanistic and in vivo evidence.

**Recent Findings:**

Fifty-two compounds from 25 primary reports encompass polyketides, nonribosomal peptides, depsipeptides, RiPPs, alkaloids, ionophores and hybrid scaffolds. Rather than a simple ranking of increasingly potent cytotoxins, the literature poses a sharper question about selectivity. Two compounds are conditionally active: kigamicin D only under nutrient deprivation and BE-43547A_2_ only under hypoxia, exploiting the metabolic state that defines the disease rather than any intrinsic difference between malignant and normal cells. Both were recovered by screens built to detect that behavior, and neither has been shown to hold a therapeutic window in vivo. Separately, thiogochangamide B retains activity in gemcitabine-resistant models, while trienomycin A binds STAT3 directly, suppresses proliferation across eight pancreatic lines, achieves 84–98% tumor growth inhibition in xenografts at doses producing no detectable toxicity, and distributes preferentially to pancreatic tissue.

**Summary:**

Conditional activity is best read as a hypothesis about how such compounds should be sought rather than as an established route to selectivity. Despite remarkable chemical diversity, translation remains constrained by the limited use of normal-cell controls, predictive biomarkers and clinically relevant preclinical models. Screens conducted in nutrient-replete, normoxic monolayer culture will systematically miss compounds of this kind. Future efforts should prioritize compounds that exploit the metabolic liabilities of pancreatic cancer rather than pursuing greater cytotoxic potency.

**Graphical abstract:**

**Figure:**
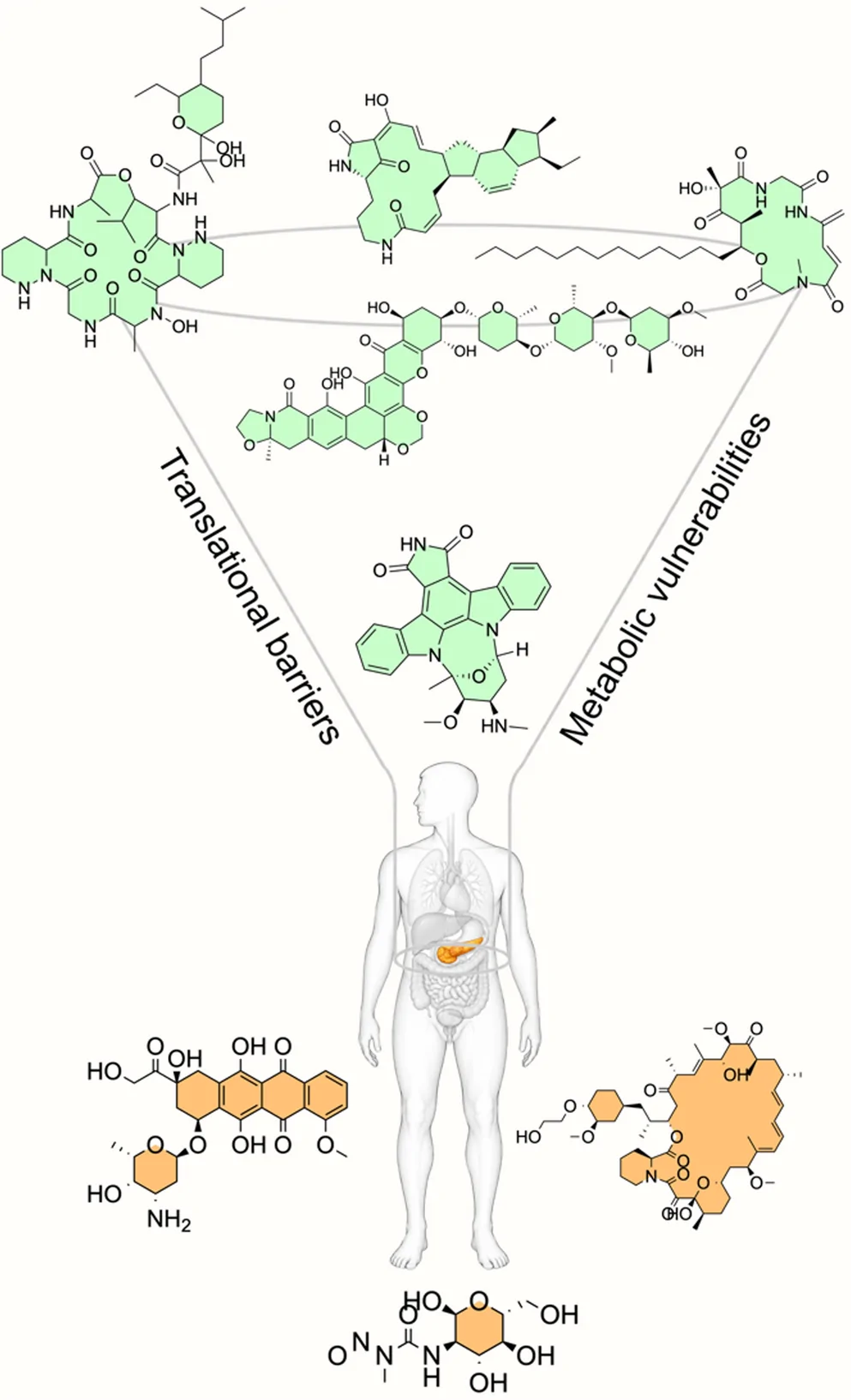

## Introduction

With a five-year survival below 13% and an incidence that continues to rise, pancreatic ductal adenocarcinoma is projected to become the second leading cause of cancer death in several countries within the decade [1–2]. It accounts for approximately 90–95% of pancreatic tumours, progresses rapidly, and responds poorly to conventional treatment [3–4]. Despite advances in combination chemotherapy regimens and the emergence of targeted and immune-based strategies, meaningful improvements in long-term survival remain elusive, underscoring the urgent need for novel therapeutic approaches [2, 4].

Natural products have historically been a major source of anticancer agents, providing structurally diverse molecules with unique and often multitarget biological activities [5–6]. Among microbial producers, *Actinomycetota* represent one of the most prolific sources of bioactive secondary metabolites, including clinically relevant drugs such as doxorubicin and actinomycin D [7]. Advances in genome mining and biosynthetic pathway analysis have further revealed that a large proportion of actinobacterial biosynthetic potential remains unexplored [8–9].

Despite the recognized biosynthetic potential of *Actinomycetota* as a source of anticancer agents, to our knowledge no review has yet compiled and critically evaluated the secondary metabolites tested specifically against pancreatic cancer models. Existing reviews have addressed either actinobacterial natural products across multiple cancer types [10–11] or pancreatic cancer therapies from a broader pharmacological perspective [2, 4], leaving a gap in the integrated analysis of chemical diversity, mechanisms of action, and translational barriers specific to this malignancy. This review addresses that gap through a critical narrative synthesis of *Actinomycetota*-derived secondary metabolites with reported activity against pancreatic cancer models, discussing their structural classes, cytotoxic and antimetastatic properties, proposed mechanisms of action, and the main challenges and perspectives for their translation into clinically relevant therapeutic strategies.

## Pancreatic Cancer: Biological Features and Therapeutic Challenges

Pancreatic cancer encompasses a heterogeneous group of malignancies arising from both the exocrine and endocrine compartments of the pancreas, of which PDAC is by far the most prevalent and clinically significant subtype [2]. A defining feature of PDAC is its highly complex tumor microenvironment, characterized by dense desmoplastic stroma, immunosuppressive cell populations, hypoxia, and nutrient deprivation, all of which collectively contribute to tumor progression, immune evasion, and intrinsic resistance to therapy [12–13].

This microenvironment is characterized by a dense desmoplastic stroma composed of extracellular matrix components, activated fibroblasts, immune cells, and abnormal vasculature [12]. Such structural and cellular complexity promotes tumor growth, facilitates metastatic dissemination, and creates physical and biochemical barriers that limit drug delivery [13]. The invasive behavior of pancreatic cancer cells further complicates disease management, as tumors frequently spread along neural and vascular structures [14]. Consequently, perineural and lymphovascular invasion are commonly observed clinicopathological features and are strongly associated with poor clinical outcomes [15].

Another challenge in pancreatic cancer management is the difficulty of early detection [2]. The disease often remains clinically silent in its initial stages, and symptoms such as painless jaundice or abdominal discomfort typically appear only when tumors have already reached advanced stages [16–17]. As a result, a substantial proportion of patients are diagnosed with locally advanced or metastatic disease, which significantly limits treatment options [4].

From a pathological perspective, pancreatic cancer commonly develops through a stepwise progression involving precursor lesions such as pancreatic intraepithelial neoplasia (PanIN) and intraductal papillary mucinous neoplasms (IPMNs), which accumulate genetic and epigenetic alterations over time [18]. These lesions represent key stages in pancreatic tumorigenesis and contribute to the molecular heterogeneity that characterizes the disease [2]. Together, these biological and clinical features make pancreatic cancer particularly difficult to treat and contribute to its poor prognosis.

## From Streptozotocin to the Present Impasse: Therapeutic Limitations and the Case for New Compounds

In 1960, a nitrosourea antibiotic was isolated from *Streptomyces achromogenes* and investigated for its antimicrobial properties. It was soon discovered instead to be a targeted cytotoxin for pancreatic β-cells, and by the early 1970s streptozotocin (STZ) was in clinical trials against islet cell carcinoma [19]. The US Food and Drug Administration approved it in 1982, and more than four decades later it remains in use, typically combined with fluoropyrimidines for treating well-differentiated pancreatic neuroendocrine tumors (panNETs).

The pharmacological basis of this success is worth stating precisely, as it defines the criteria in which the preclinical compounds surveyed later should be evaluated. STZ combines a methylnitrosourea moiety, responsible for DNA alkylation at the O6 and N7 positions of guanine, with a 2-deoxyglucose backbone that directs cellular uptake by glucose transporter type 2 (GLUT2) [19]. Its selectivity arises not from differential affinity for a tumor target but from differential delivery: cells expressing GLUT2 are markedly more sensitive than cells expressing GLUT1, and the same transporter in renal proximal tubular cells accounts for the drug’s dose-limiting nephrotoxicity. Sensitivity is further modulated by O6-methylguanine-DNA methyltransferase (MGMT), so that MGMT-deficient tumors respond better, and mismatch repair status is under investigation as a further determinant [19].

**Fig. 1:**
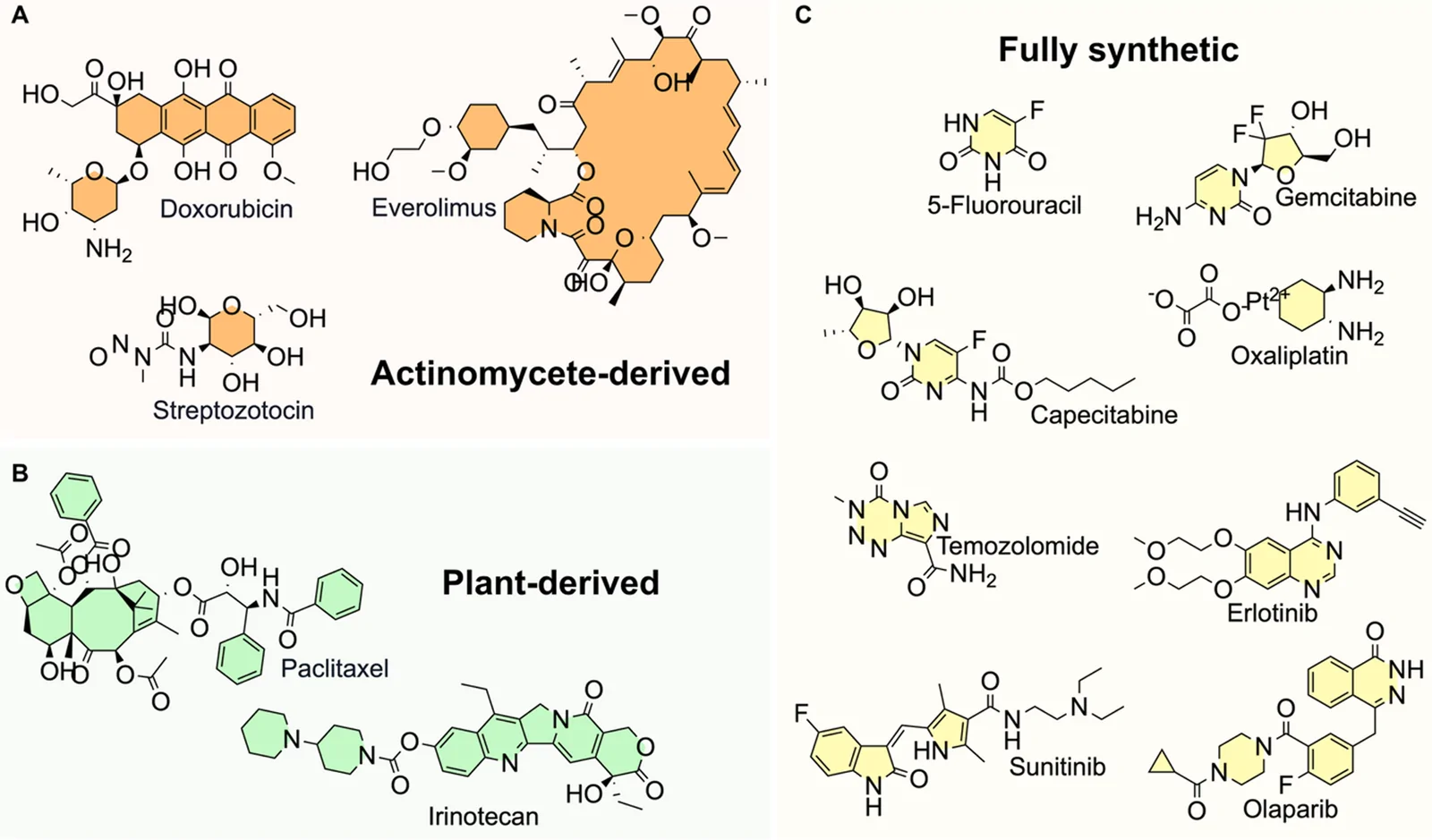
Small-molecule agents in current clinical use against pancreatic cancer, grouped by biosynthetic origin. (**A**) Actinomycete-derived agents: streptozotocin, from *Streptomyces achromogenes*; everolimus, a semisynthetic derivative of the *Streptomyces hygroscopicus* macrolide rapamycin; doxorubicin, from *Streptomyces peucetius*. (**B**) Plant-derived agents and their semisynthetic derivatives: paclitaxel, from *Taxus brevifolia*; irinotecan, a semisynthetic camptothecin from *Camptotheca acuminata*. (**C**) Fully synthetic agents: gemcitabine, 5-fluorouracil, capecitabine, oxaliplatin, temozolomide, erlotinib, olaparib and sunitinib

Everolimus, a semisynthetic derivative of rapamycin from *Streptomyces hygroscopicus*, is approved for advanced, progressive pancreatic neuroendocrine tumors, and doxorubicin, from *Streptomyces peucetius*, has been used in combination with STZ in islet cell carcinoma, where the combination outperformed STZ with 5-fluorouracil in both regression rate and survival [19]. Three of the thirteen small-molecule agents available against pancreatic malignancy therefore come from actinomycetes, a striking proportion for a single bacterial phylum, and one made visually apparent in Fig. 1.

Due to its complex biology and late-stage diagnosis, pancreatic cancer limits therapeutic options, and these are often associated with modest clinical outcomes [20]. Combination chemotherapy regimens such as FOLFIRINOX (5-fluorouracil, oxaliplatin, irinotecan, and leucovorin) or gemcitabine combined with nab-paclitaxel currently represent the standard treatment for advanced disease, offering improved survival compared with gemcitabine monotherapy [21–22]. However, these treatments frequently cause substantial toxicity and provide only incremental improvements in overall survival, which typically remains below one year for patients with metastatic pancreatic cancer [23].

The limited effectiveness of current therapies is largely explained by several intrinsic characteristics of pancreatic tumors. The dense stromal architecture restricts drug penetration, while extensive tumor heterogeneity and metabolic adaptability enable cancer cells to survive under stressed conditions such as hypoxia and nutrient deprivation [12]. These biological properties contribute to both intrinsic and acquired resistance to chemotherapy, representing major obstacles to successful treatment. Although targeted therapies, such as PARP inhibitors for BRCA-mutated tumours, have demonstrated benefits in selected patient populations, only a small proportion of patients are eligible for or can benefit from this treatment [24]. Consequently, the development of new therapeutic strategies capable of overcoming the intrinsic resistance mechanisms of pancreatic cancer remains a major priority in oncology research.

The delivery problem deserves to be stated mechanistically, because it shapes what a candidate compound must overcome. PDAC is unusual among solid tumors in being hypovascular. The neoplastic cells occupy a minority of the tumor mass, embedded in a dense matrix of collagen and hyaluronan produced largely by activated stellate cells [12–13]. Hyaluronan is strongly hygroscopic, and its accumulation raises interstitial fluid pressure to the point of collapsing intratumoral vessels, so that systemically administered drug reaches the malignant compartment poorly and unevenly [25–26]. The obvious inference, degrade the matrix and the drug will get in, has been tested clinically and has not succeeded: adding pegvorhyaluronidase alfa to nab-paclitaxel and gemcitabine failed to improve overall survival in a randomised phase III trial in hyaluronan-high metastatic PDAC [27], and depletion of myofibroblast-rich stroma in mouse models has in some settings accelerated rather than restrained disease [28]. Stromal targeting is therefore not a straightforward route to better delivery, and this qualifies the therapeutic reading of any matrix-directed natural product, including the hyaluronidase inhibitor discussed in Sect. 5.

Resistance to gemcitabine, the backbone of most regimens, is similarly well characterized and similarly instructive. The drug is a prodrug that depends on nucleoside transporters for entry and on deoxycytidine kinase for activation and is inactivated by cytidine deaminase; low expression of the transporter or of the activating kinase, and elevated ribonucleotide reductase subunits, are each associated with poor response [29]. Resistance is thus rarely the result of a mutated drug target, which is what makes it difficult to circumvent by structural analogy and what makes agents acting through unrelated mechanisms attractive.

The same properties that make these tumors resistant to conventional treatment, also impose severe constraints on the tumor cells themselves, and this is the premise of the present review. Poor perfusion means that PDAC cells thrive under oxygen tensions and degrees of nutrient deprivation that would kill most cells, sustained by KRAS-driven metabolic rewiring: increased glycolytic flux, autophagy, and macropinocytotic scavenging of extracellular protein as an amino acid source [30–32]. Each of these adaptations constitutes not only an advantage but also a dependency. A cell that survives starvation only by activating a particular survival pathway becomes, in principle, susceptible to inhibition of that pathway. Therefore, this susceptibility can in principle be exploited selectively, since normal tissue, not subjected to the same metabolic stress, does not depend on that pathway alone for survival. This reframes the search: rather than seeking compounds that are simply more cytotoxic than gemcitabine in comfortable culture conditions, one may seek compounds whose activity is conditional on the metabolic state that defines the disease. As the following sections show, several *Actinomycetota*-derived metabolites behave in exactly this way, and they were found because the assays used to look for them reproduced those conditions.

## *Actinomycetota* as a Source of Bioactive Secondary Metabolites

Within the phylum *Actinomycetota*, and particularly the genus *Streptomyces*, bacteria synthesize a myriad of bioactive secondary metabolites, such as polyketides, non-ribosomal peptides, terpenoids, alkaloids and hybrid scaffolds, and have historically been among the most productive sources of clinically relevant antibiotics and anticancer agents [7, 33–34].

Several chemotherapeutic drugs currently being used in the clinic were originated from actinobacterial metabolites. Notable examples include doxorubicin and daunorubicin, which intercalate into DNA and inhibit topoisomerase II; actinomycin D, which disrupts RNA synthesis by binding to the DNA template; bleomycin, which induces oxidative DNA strand cleavage; and mitomycin C, which acts as a DNA crosslinking agent [10, 35]. The clinical success of these compounds highlights the extraordinary capacity of *Actinomycetota* to produce molecules with precise and diverse mechanisms of action and underscores the relevance of this microbial group as a source of pharmacologically active scaffolds for oncology.

The remarkable biosynthetic capacity of *Actinomycetota* is largely explained by the abundance of biosynthetic gene clusters (BGCs) encoded in their genomes. Advances in genome sequencing and bioinformatic tools have revealed that actinobacterial genomes typically harbor dozens of BGCs. A recent large-scale analysis of over 1,000 actinomycete genomes identified more than 29,000 BGC regions, the majority of which remain cryptic or poorly characterized under standard laboratory conditions [8, 34]. These silent clusters represent an enormous reservoir of chemically unexplored natural products whose biological activities are yet to be determined.

## *Actinomycetota*-Derived Metabolites with Reported Activity Against Pancreatic Cancer

This is a narrative, critical review that compiles articles retrieved from PubMed/MEDLINE and Web of Science up to February 2026, databases that index the peer-reviewed natural-products and oncology literature most completely, using multiple Boolean permutations of terms for the disease (“pancreatic cancer”, “pancreatic ductal adenocarcinoma”), for the producing taxa (“actinomycete”, “Actinobacteria”, “Actinomycetota”, “Streptomyces”, “Micromonospora”) and for the compound class (“microbial secondary metabolite”, “natural product”). The reference lists of all retrieved articles were then screened manually (backward snowballing) until no further eligible reports were identified. We included primary reports in English that described a defined *Actinomycetota*-derived metabolite evaluated in a pancreatic cancer model, and excluded conference abstracts, reports in which neither the producing organism nor the compound was identified, and studies lacking a quantitative measure of activity. Actinomycete-derived agents that have already completed clinical development for a pancreatic indication (streptozotocin, everolimus and doxorubicin) are considered separately in Sect.  3 and Fig. 1 rather than in Table 1, which is restricted to compounds at the preclinical stage. Every potency value in Table 1 was re-derived from the primary publication and is reported in the unit in which it was originally published.

**Fig. 2:**
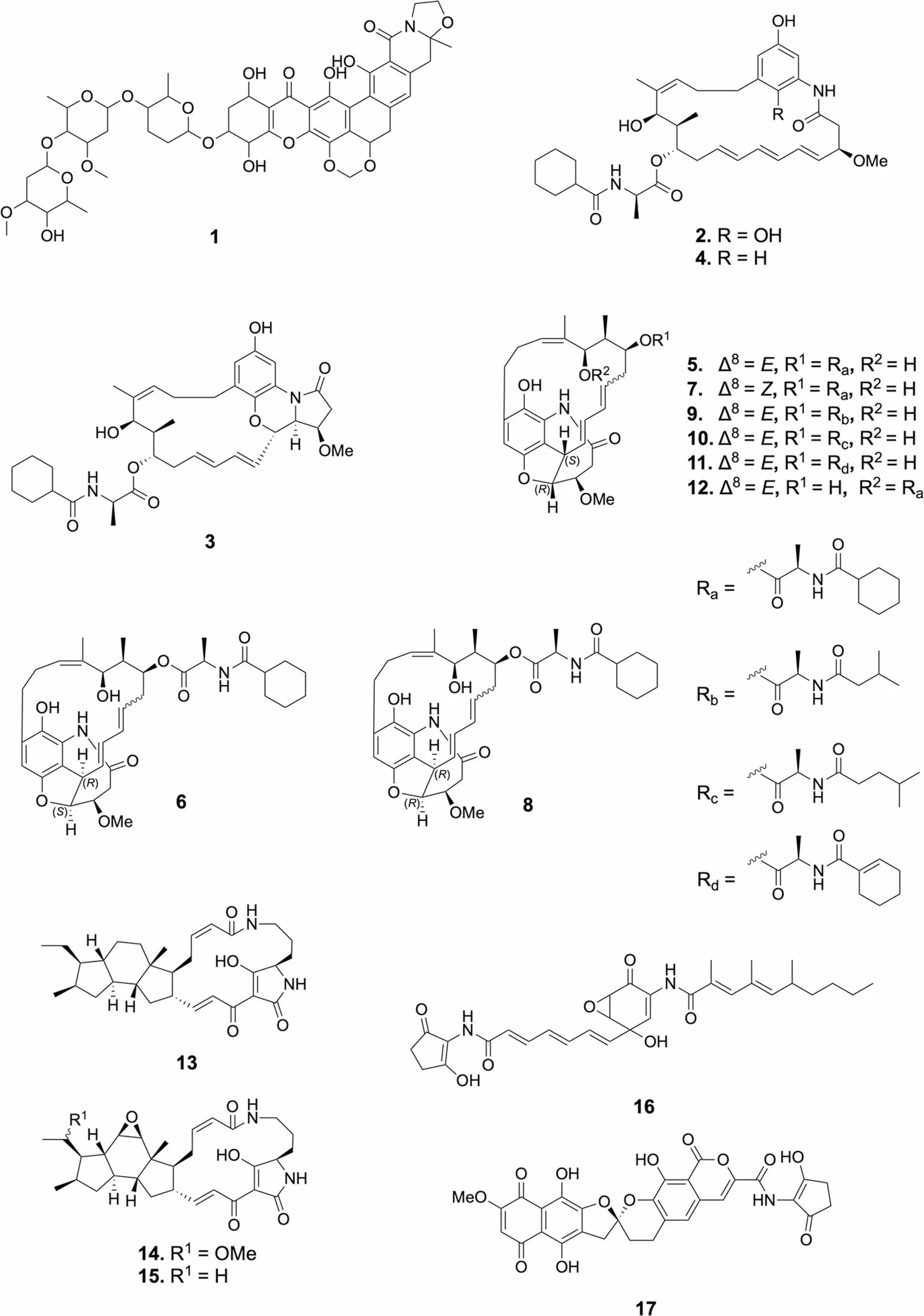
Chemical structures of the polyketides and polyketide–nonribosomal peptide hybrids. Kigamicin D (**1**); the ansatrienin-family congeners ansatrienin B (**2**), benzoxazomycin (**3**), trienomycin A (**4**) and ansafurantrienins A–H (**5**–**12**); the polycyclic tetramate macrolactams ikarugamycin (**13**), capsimycin (**14**) and capsimycin B (**15**); manumycin A (**16**); and hyaluromycin (**17**). Compound numbers correspond to those in Table 1, where producing organisms, potencies and assay conditions are given. Stereochemistry is drawn as assigned in the original isolation reports; bonds are shown without stereodescriptors where configurations were not determined

**Fig. 3:**
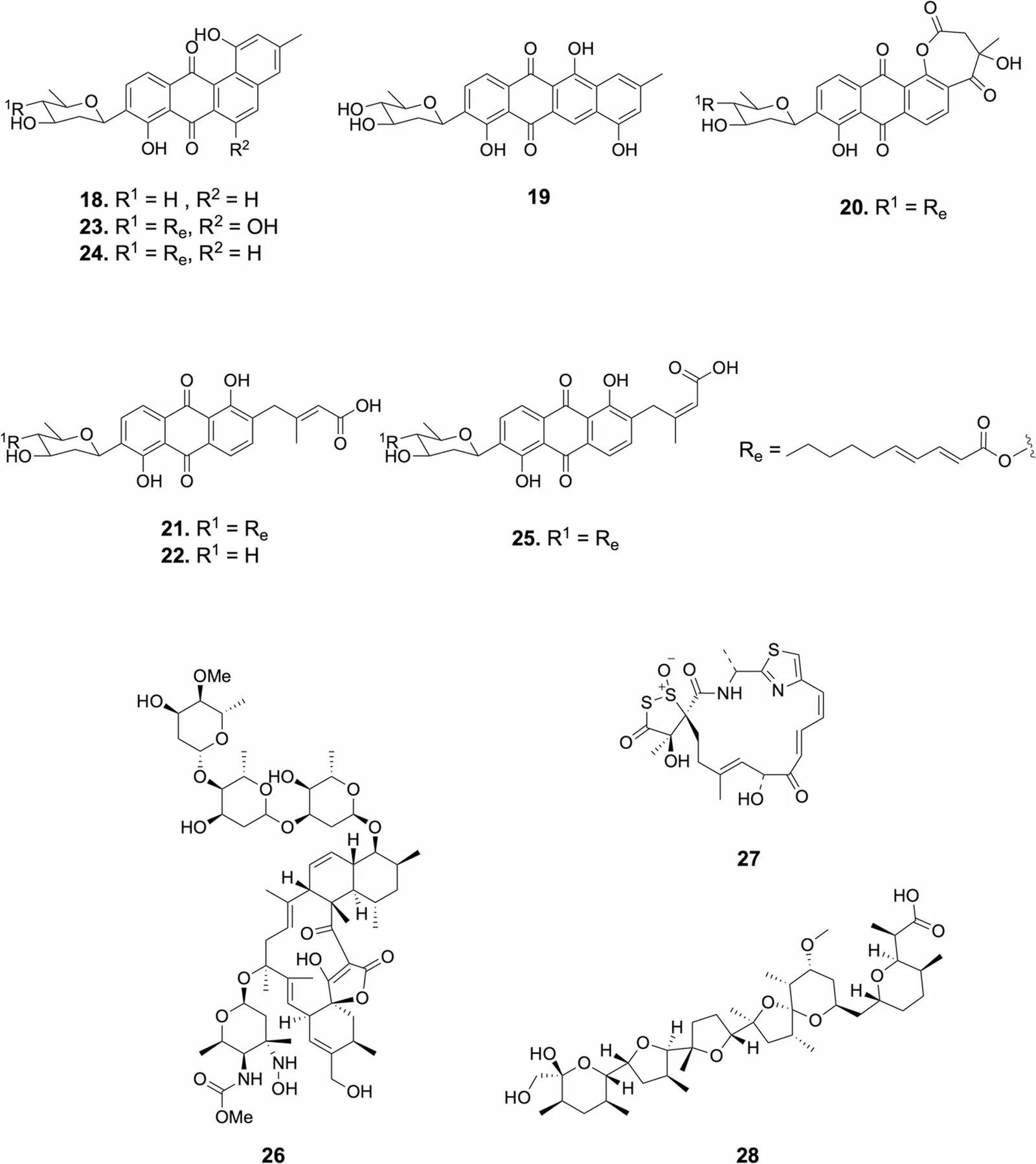
Chemical structures of the angucyclines and other polyketides. C-olivosyltetrangulol (**18**), galtamycinone (**19**) and the co-isolated congeners **20**–**25**, all from *Streptomyces* sp. P01; the spirotetronate lobophorin K (**26**); leinamycin (**27**); and the polyether ionophore nigericin (**28**). Compound numbers correspond to those in Table 1. Compounds 23–25 were inactive above 100 µg/mL and compounds 21 and 22 only weakly active (46–58 µg/mL); all five are included as structure–activity controls. Note that the two most active compounds of the series, 18 and 19, carry no seven-membered lactone, so that ring is not a general requirement for activity (Sect.  5.1)

**Fig. 4:**
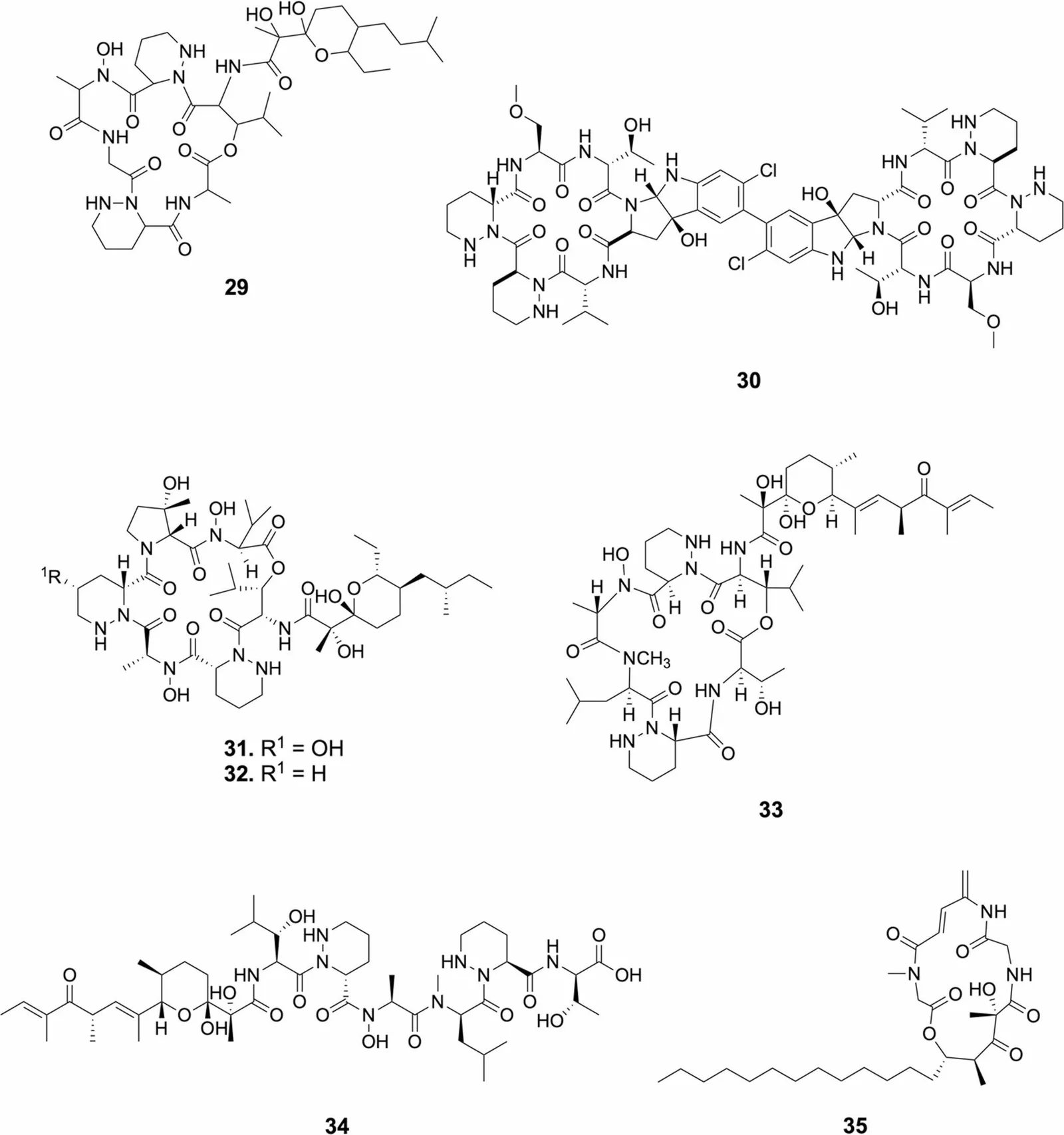
Chemical structures of the cyclic peptides and depsipeptides. Pipalamycin (**29**), chloptosin (**30**), polyoxypeptins A and B (**31**, **32**), GE3 (**33**), its linear congener GE3B (**34**) and BE-43,547 A₂ (**35**). Compounds 31–33 belong to the azinothricin family and share a macrocyclic core bearing two piperazic acid residues, differing chiefly in the acyl side chain. BE-43,547 A₂ (**35**) belongs to the 4-amino-2,4-pentadienoate-containing cyclolipodepsipeptides (APD-CLDs), together with the rakicidins. Compound numbers correspond to those in Table 1

**Fig. 5:**
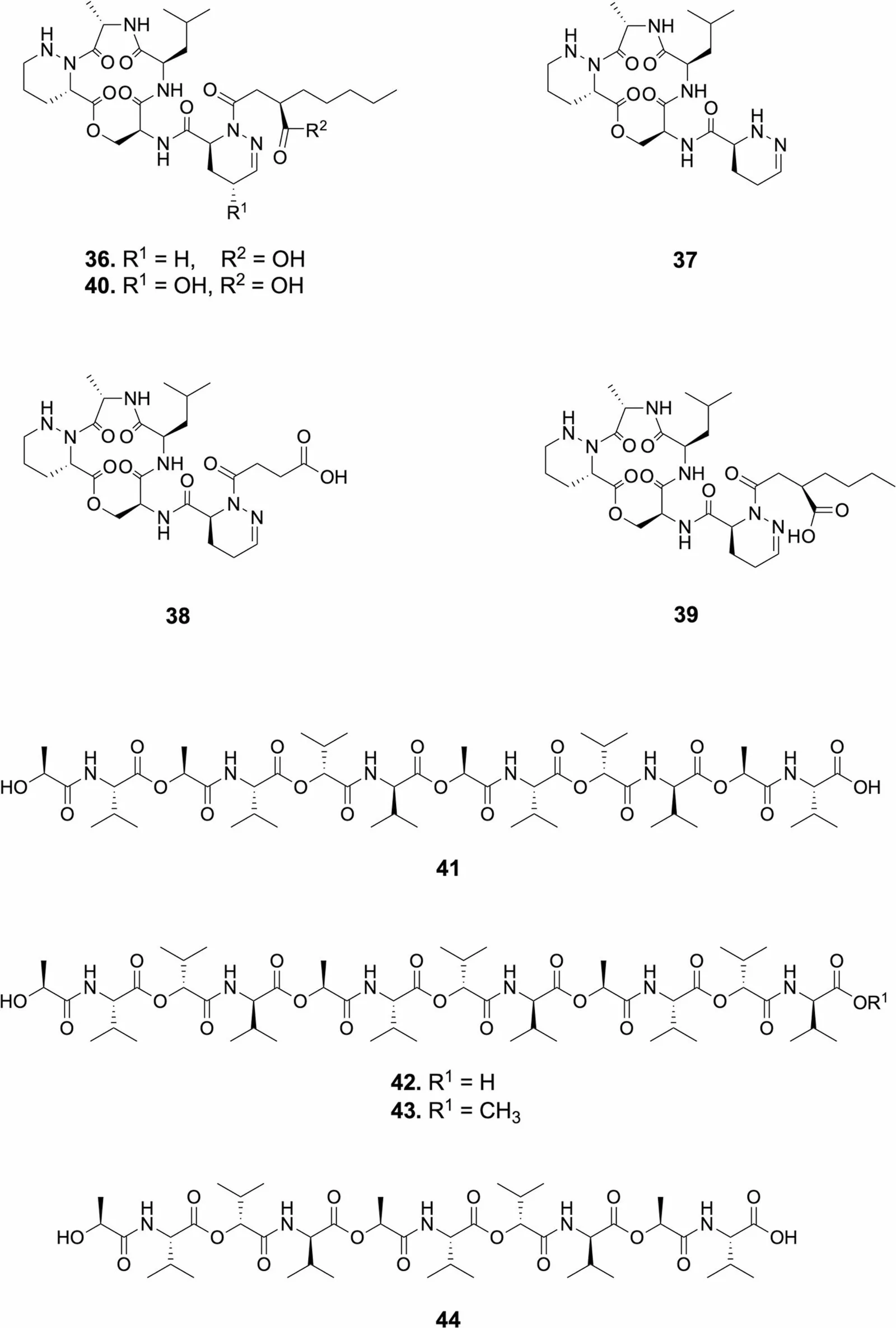
Chemical structures of the piperazic acid-bearing cyclodepsipeptides. Lydiamycins A and E–H (**36**–**40**) and of the linear oligomeric depsipeptides cavomycins A–C and the methylated derivative of cavomycin B (**41**–**44**), all discussed in Sect.  5.2. Compound numbers correspond to those in Table 1

**Fig. 6:**
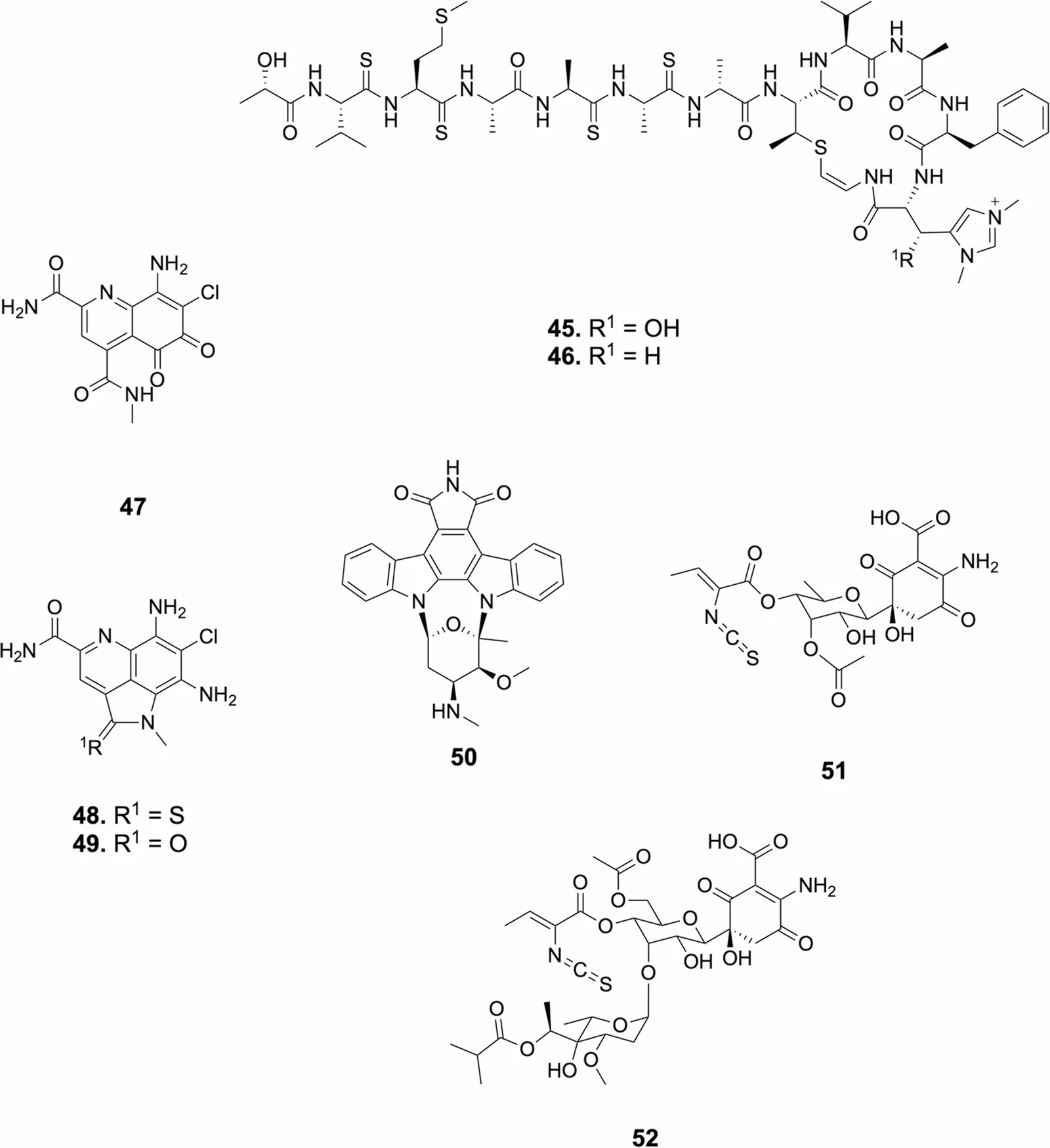
Chemical structures of the compounds discussed in Sect.  5.3, comprising ribosomally synthesised and post-translationally modified peptides, alkaloids and glycosides. The thioviridamide-family RiPPs thiogochangamides B and A (**45**, **46**); the pyrroloquinoline alkaloids ammosamides D, A and B (**47**–**49**); the indolocarbazole 7-oxostaurosporine (**50**); and the paulomycin glycosides paulomycin G (**51**) and paulomycin B (**52**), all discussed in Sect.  5.3. Compound numbers correspond to those in Table 1. Ammosamides A and B and paulomycin B were inactive in the same assays as their active congeners and are shown for comparison

The metabolites discussed below are organized according to their main structural classes, summarized in Table 1. Representative chemical structures are presented in Figs. 2, 3, 4, 5 and 6, grouped by the same biosynthetic classes used below, to facilitate structural comparison and discussion of structure-activity relationships (SAR).

**Table 1:** *Actinomycetota*-derived metabolites with reported activity in pancreatic cancer models

Compound	Producing organism	Cell line	Activity in pancreatic models*	Reference
Polyketides and polyketide-nonribosomal peptide hybrids				
Kigamicin D (1)	*Amycolatopsis sp*. ML630-mF1	PANC-1, nutrient-deprived medium	85 ng/mL (0.089 µM)	Lu et al. 2004; Kunimoto et al. 2003
Ansatrienin B (2)	*Streptomyces flaveolus* DSM 9954	PANC-1	1.04 ± 0.03 µM	Li et al. 2022
		SW1990	0.17 ± 0.01 µM	
		CFPAC-1	1.69 ± 0.07 µM	
Benzoxazomycin (3)	*Streptomyces flaveolus* DSM 9954	PANC-1	10.24 ± 0.45 µM	Li et al. 2022
		SW1990	8.29 ± 0.47 µM	
		CFPAC-1	24.32 ± 1.93 µM	
Trienomycin A (4)	*Streptomyces flaveolus* DSM 9954	PANC-1	0.80 ± 0.02 µM	Li et al. 2022
		SW1990	0.04 ± 0.01 µM	
		CFPAC-1	0.52 ± 0.02 µM	
		PANC-1	0.070 ± 0.014 µM	
		BxPC-3 (48 h / 72 h)	0.11 ± 0.010 µM	
		MIA PaCa-2 (48 h / 72 h)	0.14 ± 0.015 µM	
		CFPAC-1 (48 h / 72 h)	0.33 ± 0.040 µM	
		HPAC (48 h / 72 h)	0.39 ± 0.070 µM	
		AsPC-1 (48 h / 72 h)	0.45 ± 0.11 µM	
		Capan-2 (48 h / 72 h)	0.48 ± 0.035 µM	
		SW1990 (48 h / 72 h)	0.92 ± 0.16 µM	
Ansafurantrienin A (5)	*Streptomyces flaveolus* DSM 9954	PANC-1	3.99 ± 0.12 µM	Li et al. 2022
		SW1990	8.24 ± 0.31 µM	
		CFPAC-1	25.10 ± 1.26 µM	
Ansafurantrienin B (6)	*Streptomyces flaveolus* DSM 9954	PANC-1	17.79 ± 0.87 µM	Li et al. 2022
		SW1990	5.81 ± 0.21 µM	
		CFPAC-1	18.44 ± 0.79 µM	
Ansafurantrienin C (7)	*Streptomyces flaveolus* DSM 9954	PANC-1	9.02 ± 0.25 µM	Li et al. 2022
		SW1990	6.90 ± 0.13 µM	
		CFPAC-1	33.71 ± 1.21 µM	
Ansafurantrienin D (8)	*Streptomyces flaveolus* DSM 9954	PANC-1	4.73 ± 0.20 µM	Li et al. 2022
		SW1990	7.61 ± 0.26 µM	
		CFPAC-1	> 50 µM	
Ansafurantrienin E (9)	*Streptomyces flaveolus* DSM 9954	PANC-1	23.79 ± 1.05 µM	Li et al. 2022
		SW1990	29.77 ± 1.12 µM	
		CFPAC-1	> 50 µM	
Ansafurantrienin F (10)	*Streptomyces flaveolus* DSM 9954	PANC-1	17.59 ± 0.46 µM	Li et al. 2022
		SW1990	10.99 ± 0.44 µM	
		CFPAC-1	> 50 µM	
Ansafurantrienin G (11)	*Streptomyces flaveolus* DSM 9954	PANC-1	22.18 ± 0.98 µM	Li et al. 2022
		SW1990	34.73 ± 1.54 µM	
		CFPAC-1	> 50 µM	
Ansafurantrienin H (12)	*Streptomyces flaveolus* DSM 9954	PANC-1	7.79 ± 0.31 µM	Li et al. 2022
		SW1990	10.78 ± 0.65 µM	
		CFPAC-1	25.60 ± 0.86 µM	
Ikarugamycin (13)	*Streptomyces xiamenensis* 318	PANC-1	1.30 µM	Yu et al. 2017
Capsimycin (14)	*Streptomyces xiamenensis* 318	HPAC	2.20 µM	Yu et al. 2017
		PaTu8988	2.48 µM	
		BxPC-3	2.84 µM	
		PANC-1	3.33 µM	
		AsPC-1	7.39 µM	
		Capan-2	7.59 µM	
		CFPAC-1	10.99 µM	
		MIA PaCa-2	12.36 µM	
		HPDE-6C7	9.64 µM	
Capsimycin B (15)	*Streptomyces xiamenensis* 318	PANC-1, 24 h	3.37 µM	Yu et al. 2017
Manumycin A (16)	*Streptomyces sp*.	SUIT-2 (KRAS mut.)	3.5 µM	Kainuma et al. 1997; Ito et al. 1996
		MIA PaCa-2 (KRAS mut.)	5.0 µM	
		AsPC-1 (KRAS mut.)	6.4 µM	
		BxPC-3 (KRAS wt)	7.5 µM	
Hyaluromycin (17)	*Streptomyces hyaluromycini*	BxPC-3	No IC_50_ reported (effects at 25 and 50 µM)	Harunari et al. 2014; Kohi et al. 2016
		CFPAC-1	No IC_50_ reported (effects at 25 and 50 µM)	
C-Olivosyltetrangulol (18)	*Streptomyces sp*. P01	MIA PaCa-2	0.9 µg/mL (1.998 µM)	Ma et al. 2022
		PANC-1	5.5 µg/mL (12.21 µM)	
Galtamycinone (19)	*Streptomyces sp*. P01	MIA PaCa-2	1.1 µg/mL (2.442 µM)	Ma et al. 2022
		PANC-1	5.3 µg/mL (11.766 µM)	
Compound 5 (20) (seven-membered lactone)	*Streptomyces sp*. P01	MIA PaCa-2	1.2 µg/mL (1.897 µM)	Ma et al. 2022
		PANC-1	7.8 µg/mL (12.329 µM)	
Compound 6 (21)	*Streptomyces sp*. P01	MIA PaCa-2	49.3 µg/mL (79.686 µM)	Ma et al. 2022
		PANC-1	57.4 µg/mL (92.778 µM)	
Compound 8 (22)	*Streptomyces sp*. P01	MIA PaCa-2	46.4 µg/mL (99.048 µM)	Ma et al. 2022
		PANC-1	53.0 µg/mL (113.137 µM)	
Balmoralmycin A (23)	*Streptomyces sp*. P01	MIA PaCa-2	> 100 µg/mL	Ma et al. 2022
		PANC-1	> 100 µg/mL	
Antibiotic C104 (24)	*Streptomyces sp*. P01	MIA PaCa-2	> 100 µg/mL	Ma et al. 2022
		PANC-1	> 100 µg/mL	
Compound 7 (25)	*Streptomyces sp*. P01	MIA PaCa-2	> 100 µg/mL	Ma et al. 2022
		PANC-1	> 100 µg/mL	
Lobophorin K (26)	*Streptomyces sp*. M-207	MIA PaCa-2	34.0 ± 8.51 µM	Braña et al. 2017
		THLE-2	6.3 ± 8.2 µM	
Leinamycin (27)	*Streptomyces sp*.	MIA PaCa-2, 96 h	50 nM (0.05 µM)	Bassett et al. 2004
Nigericin (28)	*Streptomyces hygroscopicus*	PANC-1 (8 h)	39.99 ± 1.53 µM	Xu et al. 2019
		PANC-1 (16 h)	15.14 ± 1.90 µM	
		PANC-1 (32 h)	6.21 ± 2.18 µM	
		SW1990 (8 h)	25.21 ± 1.18 µM	
		SW1990 (16 h)	10.38 ± 1.62 µM	
		SW1990 (32 h)	5.79 ± 1.81 µM	
Nonribosomal peptides and depsipeptides				
Pipalamycin (29)	*Streptomyces sp*. ML297-90F8	AsPC-1 (3 d, growth)	0.03 µg/mL (0.036 µM)	Uchihata et al. 2002
		AsPC-1 (24–48 h, apoptosis)	0.3 µg/mL (0.358 µM)	
Chloptosin (30)	*Streptomyces sp*.	AsPC-1 (EC50, 24 h)	2.5 µg/mL (1.642 µM)	Umezawa et al. 2000
		AsPC-1 (IC50, 48 h)	50 ng/mL (0.033 µM)	
		BxPC-3 (IC50, 48 h)	12 ng/mL (0.008 µM)	
		HT1080 (IC50, 48 h)	5.4 ng/mL (0.004 µM)	
		Jurkat (IC50, 48 h)	6.4 ng/mL (0.004 µM)	
Polyoxypeptin A (31)	*Streptomyces sp*. MK498-98F14	AsPC-1 (IC50, 2 d)	0.062 µg/mL (0.064 µM)	Umezawa et al. 1999; Du et al. 2014
		AsPC-1 (ED50)	0.08 µg/mL (0.083 µM)	
Polyoxypeptin B (32)	*Streptomyces sp*. MK498-98F14	AsPC-1 (IC50, 2 d)	0.015 µg/mL (0.016 µM)	Umezawa et al. 1999; Du et al. 2014
		AsPC-1 (ED50)	0.17 µg/mL (0.178 µM)	
GE3 (33)	*Streptomyces sp*. GE3	HeLa S3	0.006 µM	Sakai et al. 1997
		A431	0.016 µM	
		Saos-2	0.0036 µM	
GE3B (34) (linear form)	*Streptomyces sp*. GE3	HeLa S3	> 10 µM	Sakai et al. 1997
BE-43547A2 (35)	*Streptomyces sp*.	PANC-1 — hypoxia (1% O_2_)	≈ 0.02 µM	Liu et al. 2025
		Capan-2 — hypoxia (1% O_2_)	≈ 0.10 µM	
		MIA PaCa-2 — hypoxia (1% O_2_)	≈ 0.05 µM	
		AsPC-1 — hypoxia (1% O_2_)	≈ 0.07 µM	
		PaTu8988T — hypoxia (1% O2)	≈ 0.13 µM	
		PANC-1 — normoxia	≈ 1.4 µM	
		Capan-2 — normoxia	≈ 3.6 µM	
		MIA PaCa-2 — normoxia	≈ 2.9 µM	
		AsPC-1 — normoxia	≈ 3.7 µM	
		PaTu8988T — normoxia	≈ 3.1 µM	
Lydiamycin A (36)	*Streptomyces sp*. HBQ95	PANC-1 (cytotoxicity)	> 100 µM	Wang et al. 2023
		PANC-1 (migration, 40 µM)	36% inhibition	
		PANC-1 (migration, 80 µM)	45% inhibition	
Lydiamycin E (37)	*Streptomyces sp*. HBQ95	PANC-1 (cytotoxicity)	> 100 µM	Wang et al. 2023
		PANC-1 (migration/invasion, 40–80 µM)	11–49% inhibition	
Lydiamycin F (38)	*Streptomyces sp*. HBQ95	PANC-1 (cytotoxicity)	> 100 µM	Wang et al. 2023
		PANC-1 (migration/invasion, 40–80 µM)	11–49% inhibition	
Lydiamycin G (39)	*Streptomyces sp*. HBQ95	PANC-1 (cytotoxicity)	> 100 µM	Wang et al. 2023
		PANC-1 (migration/invasion, 40–80 µM)	11–49% inhibition	
Lydiamycin H (40)	*Streptomyces sp*. HBQ95	PANC-1 (cytotoxicity)	> 100 µM	Wang et al. 2023
		PANC-1 (migration/invasion, 40–80 µM)	11–49% inhibition	
Cavomycin A (41)	*Streptomyces cavourensis*	PANC-1	> 100 µM	Wang et al. 2024
Cavomycin B (42)	*Streptomyces cavourensis*	PANC-1	1.2 µM	Wang et al. 2024
Methylated cavomycin B (2a) (43) (methylated derivative (2a)	*Streptomyces cavourensis*	PANC-1	1.7 µM	Wang et al. 2024
Cavomycin C (44)	*Streptomyces cavourensis*	PANC-1	> 100 µM	Wang et al. 2024
Ribosomal peptides, alkaloids and other classes				
Thiogochangamide B (45)	*Streptomyces sp*. GC2	PANC-1, 72 h	1.2 ± 0.17 µM	Du et al. 2026
		PANC-GR (gemcitabine-resistant)	2.9 ± 0.17 µM	
Thiogochangamide A (46)	*Streptomyces sp*. GC2	PANC-1	ND	Du et al. 2026
Ammosamide D (47)	*Streptomyces variabilis* SNA-020	MIA PaCa-2	3.2 µM	Pan et al. 2012
Ammosamide A (48)	*Streptomyces variabilis* SNA-020	MIA PaCa-2	> 20 µM	Pan et al. 2012
Ammosamide B (49)	*Streptomyces variabilis* SNA-020	MIA PaCa-2	> 20 µM	Pan et al. 2012
7-Oxostaurosporine (50) (chair conformer)	*Streptomyces sp*.	BxPC-3, KRAS wild-type	0.43 µM	Wang et al. 2020
Paulomycin G (51)	*Micromonospora matsumotoense* M-412	MIA PaCa-2	2.70 ± 0.25 µM	Sarmiento-Vizcaíno et al. 2017
		MCF-7	1.58 ± 0.12 µM	
		HepG2	4.30 ± 0.42 µM	
Paulomycin B (52)	*Micromonospora matsumotoense* M-412	MIA PaCa-2	> 36 µM	Sarmiento-Vizcaíno et al. 2017

*Potencies are given in the unit in which each source published them, with the molar equivalent in parentheses where a molecular mass is available from the primary publication or from a chemical database

### Polyketides and Polyketide-Nonribosomal Peptide Hybrids

Kigamicin D (1) is a xanthone-type polyketide identified in an anti-austerity screen designed to find compounds that kill cancer cells selectively under nutrient deprivation [36–37]. The strategy exploits a hallmark of pancreatic cancer: the capacity to survive under severe nutrient and oxygen deprivation by activation of PI3K/Akt pathway. Screening more than 2,000 actinomycete culture extracts yielded five congeners, kigamicins A–E, from *Amycolatopsis sp*. ML630-mF1, four of which (A–D) killed PANC-1 cells at concentrations a hundredfold lower under nutrient starvation than in regular culture conditions [36]. Half-maximal inhibitory concentrations were determined only for kigamicin D. Kigamicin D was preferentially cytotoxic to PANC-1 cells in nutrient-deprived medium, with an IC_50_ of 85 ng/mL, and essentially inactive in nutrient-rich medium [37]. This drug acts by blocking Akt phosphorylation induced by nutrient withdrawal, leading to cell death by necrosis [37].

Furthermore, this activity is also observed in vivo. Subcutaneous and oral administration suppressed tumor growth in nude mice xenografted with PANC-1, MIA PaCa-2 and Capan-1 cell lines [37]. Kigamicin D was subsequently shown to exhibit tumor-type selectivity, showing efficacy against certain lung xenografts but not against colorectal models, and to inhibit tumor cell-induced angiogenesis in a dorsal air sac assay [38]. The main limitation, however, is one that recurs throughout the literature, when the compound is similarly cytotoxic to nutrient-starved normal human lung fibroblasts and prostate stromal cells, indicating that its selectivity derives from the metabolic state of the cell rather than from any intrinsic distinction between tumor and host tissue [38].

Ansatrienin B (2) is a member of the ansatrienin subclass of ansamycins, 21-membered macrolactam polyketides characterized by a benzene aromatic core, a triene-containing ansa bridge, and a cyclohexanoyl-D-alanyl side chain, produced by *Streptomyces flaveolus* [39]. Across a panel of three pancreatic cancer lines the compound was active at sub- to low-micromolar concentrations (Table 1), with IC_50_ values of 1.04 ± 0.03 µM (PANC-1), 0.17 ± 0.01 µM (SW1990) and 1.69 ± 0.07 µM (CFPAC-1) [40]. Gemcitabine, assayed in parallel in the same study, gave 2.29 ± 0.08, 1.42 ± 0.03 and 0.22 ± 0.01 µM respectively, so ansatrienin B is more potent than gemcitabine in PANC-1 and SW1990 but roughly eightfold less potent in CFPAC-1. Although the precise mechanism of action of ansatrienin B against pancreatic cancer cells has not been fully elucidated, recent studies of structurally related ansatrienin analogs indicate that this compound class exerts its cytotoxic effects primarily through inhibition of signal transducer and activator of transcription 3 (STAT3) and/or the eukaryotic elongation factor 1 A (eEF1A), two targets of considerable relevance to pancreatic cancer biology [41–42]. STAT3 is constitutively activated in the majority of PDAC cases and drives tumor progression, immune evasion, and chemoresistance, while eEF1A has been implicated in cancer cell stemness and disease progression [42].

Ansafurantrienins A–H (5–12) represent a novel class of ansatrienin derivatives with unprecedented structural features, isolated from cultures of *Streptomyces flaveolus* DSM 9954 and carrying a dihydrofuran-fused benzo[b]azocine chromophore [40]. Their potencies across PANC-1, SW1990 and CFPAC-1 span roughly an order of magnitude and are given in Table 1. Two structure-activity trends are directly supported by the data. First, the intact ansatrienin core is required. Benzoxazomycin (3), a congener lacking it, was an order of magnitude less active (10.24 ± 0.45 µM in PANC-1). Second, the cyclohexanecarboxylic side chain matters more than the dihydrofuran ring itself, since the congeners bearing isobutyl, 4-methylpentyryl or cyclohexene side chains (ansafurantrienins E–G) were the weakest of the series. The most potent compound of the whole series was not an ansafurantrienin at all but the co-isolated trienomycin A (4) (0.80 ± 0.02 µM in PANC-1, 0.04 ± 0.01 µM in SW1990 and 0.52 ± 0.02 µM in CFPAC-1), which outperformed gemcitabine in PANC-1 and SW1990 but was roughly 2.4-fold less potent in CFPAC-1 (0.52 against 0.22 µM). The mechanisms underlying the activity of ansafurantrienins remain to be fully characterized. However, given the structural similarity to ansatrienin B and related ansamycins, inhibition of STAT3 and/or eEF1A cannot be excluded and warrants investigation in future mechanistic studies.

Trienomycin A is also, by some margin, the best-characterised compound of this subclass in pancreatic models. In an independent study it was recovered from a STAT3-luciferase screen of more than 1,000 natural products and shown to bind STAT3 directly by surface plasmon resonance (KD 18.0 µM), to inhibit STAT3 phosphorylation at Tyr705, and to suppress proliferation across eight pancreatic lines (Table 1) with PANC-1 the most sensitive [42]. Half-maximal inhibitory concentrations derived from those concentration–response curves span 0.20–2.42 µM at 48 h and 0.070–0.92 µM at 72 h across the eight lines (Table 1), and colony formation was suppressed at concentrations of 0.1 µM and below. The compound arrested cells in S phase, downregulated cyclin D1 and survivin, and suppressed migration and invasion without inducing appreciable apoptosis, consistent with a cytostatic rather than a cytotoxic mechanism [42].

The in vivo evidence is the strongest in this survey. In a subcutaneous PANC-1 xenograft in BALB/c nude mice, intraperitoneal administration at 10, 20 and 40 mg/kg/day produced 84%, 97% and 98% tumour growth inhibition respectively, the intermediate dose matching gemcitabine at 80 mg/kg every three days, with no significant difference in body weight between groups and no histological evidence of organ toxicity [42]. Pharmacokinetic analysis in rats showed linear kinetics after intravenous dosing and slow absorption after intraperitoneal dosing, consistent with the poor aqueous solubility of the compound, and preferential distribution to pancreatic tissue [42]. Trienomycin A is therefore the only compound surveyed here for which a route to a therapeutic window has been demonstrated, with a defined target with binding confirmed, efficacy across eight pancreatic lines, near-complete tumour growth inhibition at non-toxic doses, and tissue distribution favouring the target organ [40].

Ikarugamycin (13) is a polycyclic tetramate macrolactam (PTM) produced by mangrove-derived *Streptomyces xiamenensis* 318 [43]. PTMs are characterized by a complex multicyclic carbon framework generated through the convergence of polyketide and non-ribosomal peptide biosynthetic pathways, which confers structural rigidity and the ability to interact with diverse intracellular targets [44]. Ikarugamycin exhibits consistent antiproliferative activity against PDAC cells in the low micromolar range, with an IC_50_ of 1.30 µM against PANC-1 cells [43]. Importantly, a subsequent mechanistic study identified hexokinase 2 (HK2), the key rate-limiting enzyme of glycolysis, as a molecular target of ikarugamycin in PDAC cells [45]. Using whole-transcriptome profiling, metabolomics, surface plasmon resonance, and molecular docking, Jiang et al. (2020) demonstrated that ikarugamycin inhibits glycolytic flux in PDAC cells, as evidenced by decreased glucose-6-phosphate levels, reduced glucose consumption, and decreased lactate production and extracellular acidification rate. This mechanism is particularly relevant in the context of PDAC, where HK2 is overexpressed relative to normal tissue, is associated with poor overall survival in surgically resected patients, and drives tumor metastasis through lactate production [45]. Critically, ikarugamycin also reduced tumor size in a PDAC xenograft mouse model without overt systemic toxicity and, enhanced the chemotherapy response to gemcitabine in PDAC cells in vitro, suggesting potential as a chemosensitizer [45]. Taken together, these findings raise the translational interest of ikarugamycin and position HK2 inhibition as a promising therapeutic axis in PDAC.

Capsimycin (14) and capsimycin B (15) are PTM derivatives produced by mangrove-derived *Streptomyces xiamenensis* 318, sharing the same polyketide biosynthetic origin as ikarugamycin [43]. Capsimycin was assayed across a panel of eight pancreatic lines together with the non-tumorigenic pancreatic ductal line HPDE-6C7. IC_50_ values ranged from 2.20 µM in HPAC to 12.36 µM in MIA PaCa-2, with HPDE-6C7 at 9.64 µM [43]. Six of the eight malignant lines were therefore more sensitive than the normal cell line, but the resulting window is modest with a selectivity index of about 4.4 for the most sensitive line, HPAC, and 2.9 for PANC-1. Sensitivity across the panel did not track KRAS status. The wild-type line BxPC-3 was among the most sensitive and the KRAS-mutant MIA PaCa-2 the least. Capsimycin is biosynthetically derived from ikarugamycin through selective post-PKS modifications catalyzed by the IkaD P450 monooxygenase, which generates oxidative structural variations that confer altered target selectivity and potency [43]. Capsimycin B, formed by epoxidation catalysed by the same IkaD enzyme, gave an IC_50_ of 3.37 µM in PANC-1, a value indistinguishable from that of capsimycin (3.33 µM). The two are best described as equipotent, and a difference of this size cannot support an inference about the epoxide. The informative comparison is with ikarugamycin (1.30 µM), which retains the C-13/C-14 double bond: both epoxide-bearing congeners are roughly 2.6-fold less potent than the parent compound, indicating that epoxidation at this position is detrimental rather than neutral [43]. Notably, the same study assessed cytotoxicity towards the non-tumorigenic pancreatic ductal line HPDE-6C7 by lactate dehydrogenase release and found it negligible across the IC_50_ range of all three compounds, becoming measurable only at 10 µM [43]. The remaining congeners, capsimycins C–F (compounds 4–7 in the numbering of the original report), are terminal products of the sequential oxidative cascade catalyzed by IkaD, and were significantly less active than the first three, as judged by a pronounced reduction in inhibition ratio at 10 µM. The authors reported this as evidence for the importance of the C-13/C-14 double bond and epoxide ring, and as a possible detoxification mechanism [43].

Manumycin A (16) is a polyene polyketide of the manumycin family, built on an epoxycyclohexenone (mC7N) core with a 2-amino-3-hydroxycyclopent-2-enone (C5N) terminus, and not an ansamycin, despite being frequently described as one produced by *Streptomyces* species that exerts antiproliferative activity against multiple pancreatic cancer cell lines, including SUIT-2, MIA PaCa-2, AsPC-1, and BxPC-3 [46]. Its primary mechanism involves competitive inhibition of farnesyltransferase (FTase), the enzyme responsible for the farnesyl post-translational modification of RAS proteins, which is essential for their membrane anchoring and oncogenic activity [46–47]. This mechanism is of particular relevance to pancreatic cancer, in which activating KRAS mutations are detected in over 90% of cases [2]. The three KRAS-mutant lines were inhibited at 3.5–6.4 µM against 7.5 µM for wild-type BxPC-3, a separation no larger than the spread within the mutant group itself, and the original report states explicitly that growth was inhibited irrespective of K-ras point mutation. The KRAS dependence is confined to the downstream readouts: both MAPK activation and chemotactic invasion were suppressed selectively in the mutant background [46]. In vivo, nude mice inoculated with manumycin-pretreated SUIT-2 cells showed dose-dependent reductions in tumorigenicity and liver metastasis [46]. In BALB/c nude mice bearing MIA PaCa-2 xenografts, manumycin A at 1 and 5 mg/kg/day for five days gave T/C values of 0.79 and 0.67, and 2 mg/kg/day for two weeks reduced relative tumor volume to 1.60 ± 0.09 against 2.35 ± 0.21 in vehicle controls [47]. The effect was cytostatic rather than cytotoxic, since histology showed neither necrosis nor tumor-cell degeneration, and the bromodeoxyuridine labelling index fell only modestly. Serum aspartate aminotransferase and total cholesterol were unchanged, as was the histology of liver, kidney and heart. Tolerability was nonetheless dose-limiting outside the liver: mice receiving 5 mg/kg lost weight, reaching roughly 85% of control body weight by day 5, whereas 1 mg/kg was well tolerated [47].

These T/C values of 0.67–0.79 fall short of the conventional NCI activity threshold of T/C ≤ 0.42, and the more effective of the two doses was also the one that caused weight loss. Beyond this limited efficacy, the translational potential of manumycin A is further limited by a key resistance mechanism. Under conditions of FTase inhibition, oncogenic KRAS can undergo alternative prenylation by geranylgeranyltransferase I (GGTase-I), thereby retaining partial membrane localization and signaling capacity [46]. This compensatory mechanism has been widely observed with farnesyltransferase inhibitors and suggests that dual inhibition strategies, targeting both FTase and GGTase-I, may be required for more effective and durable KRAS pathway suppression. Furthermore, the recent development of direct KRAS inhibitors targeting specific oncogenic variants (KRAS-G12C) has significantly advanced the field, raising important questions about the comparative value of indirect prenylation inhibition strategies in KRAS-mutant pancreatic cancer [2].

Hyaluromycin (17), a rubromycin-type polyketide from marine-derived *Streptomyces hyaluromycini*, inhibits hyaluronidase with an IC_50_ of 14 µM, roughly 25-fold more potently than the reference inhibitor glycyrrhizin (340 µM) [48]. The rationale is specific to pancreatic cancer, where accelerated hyaluronan turnover generates low-molecular-weight fragments that promote proliferation, invasion, angiogenesis and chemoresistance [25–26]. In BxPC-3 and CFPAC-1 cells, hyaluromycin reduced proliferation over 1–5 days, with a marked loss of cell number at 50 µM, and inhibited transwell migration significantly at 25 and 50 µM at 24 h, a point at which proliferation was not yet affected [49]. The same treatment lowered low-molecular-weight hyaluronan in conditioned medium and decreased phosphorylation of ribosomal protein S6 at Ser235/236 in both lines, implicating indirect dampening of mTOR/PI3K signalling [49]. Targeting matrix remodelling rather than the tumor cell itself is mechanistically distinct and conceptually attractive given the desmoplastic barrier described in Sect. 2.

C-olivosyltetrangulol (18) and galtamycinone (19) are angucycline-type polyketides isolated from *Streptomyces* sp. P01, a class of polyaromatic polyketides biosynthesized by PKS type II and characterized by an angularly fused tetracyclic benz[a]anthracene scaffold [50]. Both compounds exhibited antiproliferative activity against pancreatic cancer cell lines MIA PaCa-2 and PANC-1 in the low to mid micromolar range [50]. Galtamycinone gave IC_50_ values of 1.1 µg/mL in MIA PaCa-2 and 5.3 µg/mL in PANC-1, while C-olivosyltetrangulol gave 0.9 and 5.5 µg/mL in the same two lines, against 0.68 and 1.13 µg/mL for the puromycin control assayed in parallel [50]. Both compounds are therefore about an order of magnitude less potent than the positive control, and both are appreciably more active in MIA PaCa-2 than in PANC-1. The structural diversity of angucyclines is largely driven by post-PKS tailoring reactions, including oxidative ring modifications, C- and O-glycosylation, and hydroxylation, which collectively shape their biological potency and selectivity. In particular, glycosylation patterns have been shown to profoundly influence the anticancer activity of angucyclines. C-glycosides such as C-olivosyltetrangulol exhibit distinct target interaction profiles compared to O-glycosylated analogs due to the greater conformational rigidity of the C-C glycosidic bond, which may influence binding geometry and metabolic stability [51]. While the precise mechanisms of action of these compounds against pancreatic cancer cells remain to be characterized, the demonstrated cytotoxic activity warrants further mechanistic investigation, particularly with respect to the role of the olivose sugar and the quinone moiety of galtamycinone in mediating redox-based or intercalative interactions with cellular targets.

Compound 5 (20), an angucycline derivative isolated from *Streptomyces* sp. P01, carries a seven-membered lactone ring of higher oxidation state than the parent scaffold [50]. It gave IC_50_ values of 1.2 µg/mL in MIA PaCa-2 and 7.8 µg/mL in PANC-1, against 1.1 and 5.3 µg/mL for galtamycinone. The three cytotoxic congeners of this series fall within a twofold range of one another and no meaningful potency ranking can be drawn between them [50]. Among the balmoralmycin-type congeners, only compound 5 (20), which carries the intact seven-membered lactone, was potently cytotoxic. Balmoralmycin A (23) and antibiotic C104 (24), whose A-ring is olefinic rather than lactonised, and compound 7 (25), the methyl ester of compound 6, were all inactive above 100 µg/mL, while compound 6 (21), in which the lactone is replaced by a linear 3-methylbut-2-enoic acid, and compound 8 (22), which lacks the 2,4-decadienoic acid side chain, were only weakly active (49.3 and 46.4 µg/mL in MIA PaCa-2, respectively) [50]. The lactone cannot be described as a general requirement for activity in this series, because the two most potent compounds of all, C-olivosyltetrangulol (18) and galtamycinone (19), belong to a different structural subclass and carry no seven-membered lactone at all.

Lobophorin K (26) is a glycosylated spirotetronate from *Streptomyces* sp. M-207, associated with the deep-sea coral *Lophelia pertusa*. Its reported IC_50_ against MIA PaCa-2 was 34.0 ± 85.1 µM, against MCF-7 23.0 ± 8.9 µM, and against THLE-2, an immortalised but non-tumorigenic human hepatocyte line, 6.3 ± 8.2 µM [52]. In two of these three measurements the reported standard deviation equals or exceeds the mean, so no quantitative comparison between lines can be drawn from them. What can be said is that the compound was not more active against the pancreatic line than against the normal hepatocyte or the breast carcinoma line, and that it was inactive at the maximum concentration tested against A-549, HT-29 and HepG2.

Leinamycin (27) is a hybrid polyketide-nonribosomal peptide bearing a thiazole-containing macrolactam and an unusual 1,3-dithiolan-1-oxide, and it is among the most potent compounds surveyed here, although chloptosin, polyoxypeptin B and BE-43,547 A₂ under hypoxia are more potent still in pancreatic lines. In MIA PaCa-2 cells it gave an IC_50_ of 50 nM over 96 h [53]. Its mechanism is also among the best defined. Leinamycin produced extensive DNA fragmentation and an initial arrest in S phase, and activated Chk2 but not Chk1, a pattern that points to DNA strand breaks rather than adduct formation as the proximal lesion. At higher concentrations the sub-G1 population expanded, consistent with apoptotic death secondary to DNA damage [53]. Nanomolar potency coupled with a defined DNA-damage mechanism is exactly what the field asks for, but a reactive thiol-activated alkylating agent that generates strand breaks offers no obvious basis for selectivity between tumor and normal tissue.

Nigericin (28), a carboxylic polyether ionophore, reduced viability in PANC-1 and SW1990 cells with a strongly time-dependent IC_50_ (Table 1) [54]. The same study characterised circular RNA expression profiles after treatment by high-throughput sequencing, but no molecular target was identified and the reported circRNA–miRNA–mRNA networks are computational predictions rather than validated mechanism.

### Nonribosomal Peptides and Depsipeptides

Pipalamycin (29) is a cyclic hexadepsipeptide isolated from *Streptomyces* sp. ML297-90F8, composed of alanine, N-hydroxyalanine, glycine, N-acylated 3-hydroxyleucine, and two residues of piperazic acid [55]. Its most clinically relevant property is the ability to induce apoptosis in AsPC-1 human pancreatic adenocarcinoma cells, a cell line well documented for its resistance to apoptotic stimuli. Pipalamycin treatment over 72 h inhibited AsPC-1 cell proliferation with an IC_50_ of 0.03 µg/mL [55]. The capacity to overcome intrinsic apoptosis resistance matters in PDAC, where evasion of programmed cell death frequently drives treatment resistance [56]. However, the precise molecular mechanism by which pipalamycin triggers apoptosis in these resistant cells has not been fully elucidated [55].

Chloptosin (30) is a dimeric cyclohexapeptide of unusual dumbbell-type architecture, isolated from a *Streptomyces* culture broth in a screen for apoptosis-inducing agents [57]. Its structure consists of two cyclohexapeptide units, each containing D-valine, (3 S)- and (3R)-piperazic acids, O-methyl-L-serine, D-threonine, and the non-proteinogenic residue (2 S,3aR,8aR)-6-chloro-3a-hydroxy-2,3,3a,8a-hexahydropyrrolo[2,3-b]indole-2-carboxylic acid, with amino acid components arranged in alternating stereochemical configurations [57]. Like pipalamycin, chloptosin was identified by its ability to induce apoptosis in apoptosis-resistant AsPC-1 pancreatic adenocarcinoma cells at low concentrations. Chloptosin reduced cell viability in AsPC-1 cell line with an EC_50_ of 2.5 µg/mL at 24 h, and inhibited proliferation of the same line with an IC_50_ of 50 ng/mL at 48 h, with corresponding growth-inhibitory values of 12 ng/mL in BxPC-3 cell line [57]. It is worth noting that AsPC-1, the apoptosis-resistant cell line, was the least sensitive of the four lines tested. Mechanistically, the death chloptosin induces is caspase-independent and is accompanied by selective inhibition of RNA synthesis [57].

Polyoxypeptins A (31) and B (32) were isolated from *Streptomyces* sp. MK498-98F14 [58]. They are cyclic hexadepsipeptides carrying an acyl side chain and two piperazic acid units, placing them in the azinothricin family alongside GE3, the lydiamycins and A83586C. Both inhibited proliferation of the apoptosis-resistant AsPC-1 line with IC_50_ values of 0.062 and 0.015 µg/mL respectively, and decreased viability with ED_50_ values of 0.08 and 0.17 µg/mL [58]. Adriamycin and vinblastine, tested in parallel, failed to kill AsPC-1 cells even at 30 µg/mL [58]. The polyoxypeptin A biosynthetic gene cluster has since been characterised, identifying a hybrid NRPS–PKS assembly line together with the enzymes that supply its unusual (2 S,3R)-3-hydroxy-3-methylproline and piperazic acid building blocks [59], which makes this one of the few compounds in the present survey for which engineered analogue production is a realistic prospect.

GE3 (33) is a cyclic hexadepsipeptide from *Streptomyces* sp. GE3. It is extraordinarily cytotoxic, with IC_50_ values of 3.6 nM in Saos-2, 6 nM in HeLa S3 and 16 nM in A431 cells. However, the same study reported an IC_50_ of 7 nM in BALB/3T3, a non-transformed murine fibroblast line, giving a selectivity index of approximately one [60]. Against PSN-1 pancreatic carcinoma xenografts GE3 gave a T/C of 0.47 after a single intraperitoneal dose of 2 mg/kg, above the conventional NCI activity threshold of 0.42, and that dose caused a loss of 18.2% of body weight [60]. Its linear congener GE3B (34) was inactive both as an antibiotic and as a cytotoxin, confirming that the macrocycle is required.

BE-43,547 A₂ (35), a 4-amino-2,4-pentadienoate-containing cyclolipodepsipeptide (APD-CLD) from *Streptomyces* sp., is the clearest example in this survey of selectivity conferred by tumor physiology rather than by molecular structure. Across five pancreatic cancer lines (PANC-1, Capan-2, MIA PaCa-2, AsPC-1 and PaTu8988T) its IC_50_ under hypoxia was consistently and substantially lower compared to normoxia [61]. Hypoxic IC_50_ can be estimated at roughly 0.02–0.13 µM against 1.4–3.7 µM under normoxia. The ratio ranges from about 24-fold in PaTu8988T to about 70-fold in PANC-1. Proteomics identified the eukaryotic elongation factor eEF1A1 as the direct binding partner in PaTu8988T cells under hypoxia, and target engagement was confirmed by cellular thermal shift assay in PANC-1 lysate [61]. In vivo efficacy was demonstrated in a PANC-1 xenograft in nude mice, but using NMP-BE-43,547 A₂ citrate, a semisynthetic prodrug prepared to overcome the poor aqueous solubility of the natural product [61]. Because hypoxia is a defining feature in the PDAC tumor microenvironment described in Sect. 2, and because it represents precisely the condition under which conventional chemotherapy fails, a compound whose potency increases rather than diminishes under these conditions is of particular interest. The same hypoxia-dependent potency has, however, been reported for this compound in glioblastoma and lung cancer lines [61], so the behaviour is a general property of the molecule rather than a pancreas-specific one.

Lydiamycins A (36) and E–H (37–40) are piperazic acid-bearing cyclodepsipeptides from an endophytic *Streptomyces* sp. associated with *Cinnamomum cassia*, and their pharmacological profile is categorically different from that of the compounds above [62]. None was cytotoxic to PANC-1 at 100 µM. All nonetheless suppressed migration and invasion in scratch-wound and Transwell assays, by 11–49% at 40–80 µM and without morphological signs of cell death, with lydiamycin A the most active and exceeding 5-fluorouracil in the invasion assay.

Cavomycins A (41), B (42), its methylated derivative (43) and C (44) are linear oligomeric depsipeptides produced by *Streptomyces cavourensis* HK58, an actinomycete isolated from the gut microbiota of the marine annelid *Paraleonnates uschakovi* [63]. Cavomycin B and its methylated derivative were active in the low micromolar range, with IC_50_ values of 1.2 µM and 1.7 µM respectively, while cavomycins A and C both showed IC_50_ values exceeding 100 µM, indicating minimal cytotoxic activity in PANC-1 cells [63]. Methylation of cavomycin B shifted the IC_50_ from 1.2 to 1.7 µM. Neither mechanism nor in vivo efficacy has been investigated.

### Ribosomal Peptides, Alkaloids and Other Classes

Thiogochangamides B (45) and A (46) were discovered by a metabologenomic strategy that used the TfuA-encoding gene as a genomic hallmark of thioamide biosynthesis and the isotopic signature of sulfur as a metabolomic one [64]. They belong to the thioviridamide family and are therefore ribosomally synthesised and post-translationally modified peptides (RiPPs). Thiogochangamide B gave an IC_50_ of 1.2 ± 0.17 µM in PANC-1 and 2.9 ± 0.17 µM in PANC-GR, a gemcitabine-resistant derivative of the same line, against 2.1 ± 0.05 µM and more than 50 µM for gemcitabine assayed in parallel [64]. The resistance ratio is therefore about 2.4-fold for the natural product and more than 24-fold for the clinical drug. Mechanistically it downregulated Wnt/β-catenin signalling, reduced nuclear β-catenin, and suppressed migration, invasion and the epithelial–mesenchymal transition markers (N-cadherin, MMP7, Snail) in PANC-GR cells. In a PANC-GR cell-line-derived xenograft, 1 mg/kg intraperitoneally three times weekly for 20 days reduced tumor volume and weight without weight loss or overt toxicity, and combination with gemcitabine was synergistic by the Chou–Talalay method [64]. This is the only compound in the present survey shown to overcome acquired resistance to the standard of care in both cell and animal models.

Ammosamide D (47), a pyrroloquinoline-type alkaloid isolated from *Streptomyces variabilis* SNA-020, exhibits moderate antiproliferative activity against pancreatic cancer cells, with an IC_50_ of 3.2 µM in the MIA PaCa-2 model [65]. Its mechanism is proposed to involve covalent binding to cellular proteins, potentially disrupting essential processes related to cytoskeletal organization and cell viability [65–66]. The comparison with the parent congeners points in the opposite direction, since neither ammosamide A (48) nor ammosamide B (49) was cytotoxic to MIA PaCa-2 below 20 µM, whereas the oxidatively ring-opened congener ammosamide D was active at 3.2 µM [65], .

7-Oxostaurosporine (50) is an indolocarbazole alkaloid isolated from a marine-derived *Streptomyces* sp. that functions as a dual inhibitor of protein kinase C theta (PKCθ, IC_50_ 6 nM) and delta (PKCδ, IC_50_ 21 nM) [67]. Indolocarbazole denotes the structural class rather than an individual compound. The congener responsible for the activity described below is the chair conformer of 7-oxostaurosporine. PKC isoforms are aberrantly activated in multiple human cancers, including PDAC, where they promote tumor cell survival, growth, and chemoresistance through activation of downstream survival signaling pathways [67]. 7-Oxostaurosporine exhibits potent antiproliferative activity against BxPC-3 pancreatic cancer cells, with an IC_50_ of 0.43 µM [67]. BxPC-3 carries wild-type KRAS, which is worth noting alongside the KRAS-dependent mechanisms discussed above. SAR studies of indolocarbazole derivatives reveal that the planar, fused indolocarbazole core scaffold is essential for PKC inhibition and that peripheral substituents tune both potency and isoform selectivity [67]. These conclusions rest on the largest structure–activity campaign in the whole of this literature, since 69 natural indolocarbazoles were isolated from marine-derived actinomycetes, 15 of them previously undescribed, and all were assayed against PKCθ and analysed by 3D-QSAR. The most striking result of that series is conformational rather than constitutional. The chair conformer of 7-oxostaurosporine inhibited PKCθ far more potently than the corresponding boat isomer. Importantly, 7-oxostaurosporine demonstrated in vivo efficacy in a BxPC-3 xenograft model in BALB/c nude mice at 6 mg/kg/day intravenously, achieving tumor growth inhibition of 49.6% without overt signs of systemic toxicity, and of 56.9% in a separate patient-derived xenograft model of pancreatic cancer [67]. Together with thiogochangamide B and trienomycin A, this is among the most advanced translational evidence in the set surveyed here, since a patient-derived xenograft preserves the stromal and genetic features that monolayer culture discards. This activity validates the translational potential of PKCθ/δ inhibition as a therapeutic strategy in PDAC and positions 7-oxostaurosporine as a promising lead compound for further development.

Paulomycin G (51), from deep-sea sediment-derived *Micromonospora matsumotoense* M-412 recovered at 2,000 m in the Avilés Canyon, is the first paulomycin lacking the paulomycose sugar and the smallest bioactive member of the family [68]. Against MIA PaCa-2 it gave an IC_50_ of 2.70 ± 0.25 µM, but comparable potency against MCF-7 (1.58 ± 0.12 µM) and HepG2 (4.30 ± 0.42 µM) points to general cytotoxicity rather than pancreas-directed selectivity. Paulomycin B (52), tested in parallel, was inactive against all three lines above 36 µM, a genuine structure–activity contrast, unlike several of the marginal comparisons made elsewhere in this literature. No in vivo efficacy studies of paulomycin G in pancreatic cancer models have been reported. However, given its potent cytotoxicity in vitro and its unique structural features, further investigation of its mechanism(s) of action and in vivo efficacy in pancreatic xenograft models would be highly warranted.

## Challenges and Perspectives

*Actinomycetota* have long been recognized as one of the most prolific sources of structurally diverse secondary metabolites with antitumor potential, and recent reviews covering the period from 2019 to 2024 have confirmed that this biosynthetic capacity extends across a wide spectrum of cancer types [69–70]. The compounds surveyed here extend this evidence to the specific context of pancreatic cancer, collectively demonstrating that *Actinomycetota-*derived metabolites can interfere with a broad range of biological processes relevant to PDAC, including proliferation, apoptosis, metabolic reprogramming, extracellular matrix remodeling, and cell migration. What this survey suggests is narrower: the phylum supplies scaffolds of the right potency range and, in a minority of cases, of defined mechanism, but the pharmacological work needed to tell a lead from a general cytotoxin has largely not been done.

A fundamental limitation shared by most of these studies is the incomplete characterization of primary molecular targets. Antiproliferative activity was established almost everywhere through cytotoxicity assays alone, without the mechanistic work needed to distinguish pathway-specific modulation from generalized toxicity. Without defined targets it is largely speculative to optimize pharmacokinetics, predict selectivity, minimize off-target effects or rationalize SAR [71–72]. In the context of PDAC specifically, target-based approaches are further complicated by the scarcity of fully validated drug targets that are not confounded by the tumor microenvironment [14]. Addressing this gap will require the adoption of integrated chemoproteomics strategies, including activity-based protein profiling (ABPP), thermal proteome profiling (TPP), and drug affinity-responsive target stability (DARTS), capable of identifying direct protein targets in biologically relevant cellular systems [71].

A second major challenge concerns the biological models used to assess activity. Most studies reviewed relied on conventional two-dimensional cell line cultures, which fail to recapitulate the three-dimensional spatial architecture, cellular heterogeneity, and stromal interactions that are defining features of PDAC [73]. Patient-derived organoids (PDOs) have emerged as particularly compelling alternatives, since they preserve the genetic and morphological characteristics of the primary tumor, exhibit over 91% predictive accuracy for patient responses to first-line chemotherapies [73–74]. Incorporating PDAC organoid platforms into the preclinical evaluation of *Actinomycetota*-derived metabolites or other natural products would substantially enhance the translational relevance of future bioactivity data, enabling drug response profiling under conditions that more accurately reflect the disease biology.

A third dimension is the potential of compounds that modulate cell migration and invasion without inducing direct cytotoxicity. PDAC is predominantly a lethal disease because of metastasis, since most patients are diagnosed with locally advanced or metastatic disease, and active metastatic seeding from both primary and secondary sites continues throughout disease progression [10, 75]. The concept that migrating, invasive cells may be less sensitive to cytotoxic therapies, a phenomenon related to the mutual exclusivity of proliferation and migration known as the “go or grow” effect, provides a biological rationale for pursuing antimetastatic agents as a complementary therapeutic strategy [75]. Within the compounds reviewed here, lydiamycins and hyaluromycin exemplify this profile, and their further mechanistic investigation is warranted in this specific context.

The most productive way to read this body of work, however, is not compound by compound but along the axis that several of the best-evidenced examples share. Kigamicin D is essentially inactive in nutrient-rich medium and cytotoxic only under nutrient deprivation; BE-43547A2 is an order of magnitude more potent under hypoxia than normoxia; ikarugamycin acts on hexokinase 2 and suppresses glycolytic flux; and streptozotocin, the only member of this chemical universe approved for a pancreatic malignancy, owes its tissue tropism to uptake through GLUT2 rather than to any differential affinity for a tumor target. In each case selectivity resides in the metabolic state of the tumor or in the transporters that serve it, not in the molecule. This convergence is unlikely to be coincidental. Nutrient deprivation, hypoxia and glycolytic dependence are defining features of the desmoplastic, poorly perfused PDAC microenvironment described in Sect.  2, and they are the same features that blunt conventional cytotoxic chemotherapy. A compound whose potency rises rather than falls under those conditions inverts the usual pharmacological problem.

Framing the field this way has two practical consequences. The first concerns how activity is measured: a screen conducted in nutrient-replete, normoxic monolayer culture will systematically miss compounds of this kind and will report as inactive precisely the molecules whose selectivity depends on stress. The second concerns biomarkers. Streptozotocin shows that a cytotoxic agent of unremarkable intrinsic potency can retain clinical utility for five decades when a predictive marker, MGMT deficiency, identifies the patients likely to respond. None of the preclinical metabolites surveyed here has a candidate biomarker of this kind. Given how many of them sit in the same low-micromolar band, identifying which tumors depend on the vulnerability a given compound exploits may matter more for translation than a further increment in potency.

Finally, this review has limitations of its own that bear on how its conclusions should be read. The primary literature it draws on is also heterogeneous in ways that resist synthesis. Potency values originate from different assay formats and exposure times, from 8 h in the case of nigericin to 96 h for leinamycin, and, as noted for nigericin, the same compound and cell line can differ more than sixfold across exposure times. Trienomycin A, assayed by two independent laboratories under a nominally shared protocol, differs between them by up to sixtyfold [40, 42], so potency values from different sources are not necessarily commensurable even where units and cell lines agree. Normal-cell controls are reported in only a minority of studies, and a clinical comparator such as gemcitabine in fewer still, which means that most of the selectivity indices a reader would want cannot be calculated. Publication bias probably compounds this, since compounds that proved inactive in pancreatic models are unlikely to have been reported. The reader should therefore treat Table 1 as a map of where work has been done rather than as a ranking of therapeutic promise.

## Concluding Remarks

Are *Actinomycetota* a good place to look for compounds against pancreatic cancer? On the evidence assembled here the answer is a qualified yes, but not for the reason usually given. Potency is not the argument, since the metabolites surveyed cluster in the same low-micromolar band as many synthetic leads, and where a normal-cell comparator exists the selectivity indices are modest. That criticism, however, should not be pressed too far, since it applies with equal force to most drugs in current use: doxorubicin, cisplatin and gemcitabine are not selective cytotoxins either, and their therapeutic index derives from pharmacokinetics, scheduling and tissue distribution rather than from differential killing in culture. The correct question is therefore not whether a compound spares normal cells in a dish, but whether there is a principled route to a therapeutic window in vivo. Judged that way, this chemical space performs reasonably well. It has yielded compounds with defined targets (hexokinase 2 for ikarugamycin, eEF1A1 for BE-43547A2, PKCθ/δ for 7-oxostaurosporine, Wnt/β-catenin for thiogochangamide B, farnesyltransferase for manumycin A, Akt for kigamicin D, STAT3 for trienomycin A) and, more distinctively, compounds whose activity is conditional on the metabolic state that defines the disease. Kigamicin D acts only under nutrient deprivation and BE-43,547 A₂ only under hypoxia. These two compounds are the whole of the direct evidence, and both were recovered by screens built to detect precisely that behaviour, so the pattern may reflect how they were sought as much as how the chemistry behaves. Conditional activity is nonetheless the most interesting hypothesis this literature has produced, and the one that bears most directly on how future screens in PDAC should be designed. It remains a hypothesis. No compound of this class has been shown to hold a therapeutic window in vivo, and agents directed at hypoxia and tumour metabolism have a poor clinical record in PDAC evofosfamide, which failed to improve survival in a randomised phase III trial, being the clearest example.

It is worth noting that the compound with the strongest dossier assembled here does not owe it to conditional activity at all. Trienomycin A acts on a conventional oncogenic target, STAT3, and its apparent therapeutic window rests on ordinary pharmacology — tissue distribution that favours the pancreas, and efficacy at doses well below those producing toxicity. That is precisely the basis on which the therapeutic index of established cytotoxics rests, and it suggests that the two routes to selectivity discussed here are complementary rather than competing: conditional activity offers a way to exploit the metabolic state of the tumour, while favourable distribution offers a way to reach it.

The spread of that list should not be overstated. Four of the six are kinases or kinase-dependent pathways, and two of the targets, farnesyltransferase and PKC, were the object of extensive rational-design campaigns, so most of this list is not territory that microbial chemistry reached first. eEF1A1 is the clearest exception, a target synthetic libraries have rarely delivered, though it recurs here rather than standing alone, having also been proposed for the ansatrienins. What it has not yet done is validate them — most rest on a single study — and that, rather than the search for further molecules, is where the field’s effort now belongs.

## Data Availability

No datasets were generated or analysed during the current study.
